# Tenapanor as Add-on Treatment for Hyperphosphatemia in Dialysis Patients: Enough Bang for the Buck?

**DOI:** 10.1016/j.ekir.2023.10.001

**Published:** 2023-10-06

**Authors:** Jay B. Wish

**Affiliations:** 1 Division of Nephrology, Indiana University School of Medicine, IU Health University Hospital, Indianapolis, Indiana, USA

See Clinical Research on Page 2243

Management of hyperphosphatemia in patients on dialysis is often challenging. The Kidney Disease Improving Global Outcomes 2017 Clinical Practice Guideline Update for the Diagnosis, Evaluation, Prevention and Treatment of Chronic Kidney Disease-Mineral and Bone Disorder recommends that treatment of chronic kidney disease-mineral and bone disorder should be based on serial assessments of phosphate, calcium, and parathyroid hormone considered together (4.1.1); lowering elevated phosphate levels toward the normal range (4.1.3), and restricting the use of calcium-based phosphate binders (4.1.6).^1^ These recommendations provide a more nuanced approach to chronic kidney disease-mineral and bone disorder than the 2009 guideline, acknowledging that interventions to correct one chronic kidney disease-mineral and bone disorder biochemical parameter may have adverse consequences on another which blunts the clinical benefit. The recommendations are based on substantial observational data linking hyperphosphatemia with adverse clinical outcomes; however, there are no randomized clinical trials demonstrating that correction of hyperphosphatemia leads to improved patient outcomes. Elevated serum phosphorus levels induce multiple physiological abnormalities associated with increased risk of morbidity and mortality from cardiovascular disease, including endothelial dysfunction leading to cell apoptosis, calcification of vascular smooth muscle cells, and increases in fibroblast growth factor-23 and parathyroid hormone levels.^2^ Accordingly, reduction of serum phosphorus to a level close to the normal range has become a standard-of-care for patients on dialysis, and is an internal performance metric for many dialysis providers. In February 2021, data from the US Dialysis Practice and Patterns Study showed that only 55.7% of 8654 patients in the stratified random sample had a 3-month average serum phosphorus level ≤5.5 mg/dl.^3^

Serum phosphorus reduction can be achieved by dietary phosphorus restriction, removal of phosphorus by dialysis, and/or the use of orally administered medications. Dietary phosphorus restriction is difficult because of the widespread presence of phosphorus in foods, especially as an additive in packaged products to increase shelf life, fast foods, restructured meats, processed cheeses, “instant” puddings and sauces, and soft drinks. Phosphate removal on dialysis is more dependent on the duration of the dialysis treatment than on dialysis frequency. The Frequent Hemodialysis Network Nocturnal Trial^4^ demonstrated that patients randomized to 6 times weekly nocturnal dialysis for an average of 6.3 hours per session achieved an average serum phosphorus of 4.08 mg/dl, most without the use of phosphate binder medications. Short daily hemodialysis has not been shown to produce greater reductions in serum phosphorus than conventional 3 times weekly hemodialysis. Continuous ambulatory peritoneal dialysis leads to greater phosphate removal than automated peritoneal dialysis due to the longer dwell period of the former.S1 Hemodiafiltration provides 10% greater phosphate removal than a comparable hemodialysis session.S2 Phosphate binder medications have been the mainstay of treatment of hyperphosphatemia. A variety of these medications are available, each with characteristics such as cost, pill burden, side effects, and chew versus swallow-whole that may affect patient adherence. What all phosphate binders have in common is that they must be administered with meals in order to bind the phosphate in ingested food. Many patients report this decreases their enjoyment of the meal, and patients who eat outside the home may forget to bring their phosphate binder medication with them. The lack of success with current approaches to lowering serum phosphorus levels provided an unmet need for an intervention with a different mechanism of action and dosing schedule that may improve patient adherence and efficacy.

Tenapanor is a novel phosphate lowering medication that inhibits the sodium/hydrogen exchange isoform 3 in the gastrointestinal tract. Because it acts on phosphate transport by the gastrointestinal tract and not as a binder, it does not have be taken with meals, and that may increase adherence. In the first phase 3 trial published in 2019,^5^ 219 subjects treated with tenapanor 3 mg twice daily (bid), 10 mg bid, or 30 mg bid for 8 weeks sustained a reduction in serum phosphorus of about 1 mg/dl from baseline. During a 4-week withdrawal period versus placebo, patients continued on tenapanor maintained stable serum phosphorus levels whereas those switched to placebo increased serum phosphorus by a mean of 0.79 mg/dl. In the AMPLIFY trial,^6^ 236 patients undergoing maintenance dialysis with hyperphosphatemia despite phosphate binder therapy were randomized to tenapanor or placebo for 4 weeks and continued on their baseline dose of phosphate binder. Subjects receiving tenapanor sustained a reduction in serum phosphorus from baseline of 0.84 mg/dl versus 0.19 mg/dl for those randomized to placebo. Diarrhea was the most reported adverse event (42.7% for tenapanor vs. 6.7% for placebo), resulting in drug discontinuation in 3.4% and 1.7% of subjects, respectively. In the PHREEDOM trial^7^, 564 participants undergoing maintenance dialysis with both hyperphosphatemia (serum phosphate 6.0–10.0 mg/dl) on phosphate binders and a 1.5 mg/dl increase after phosphate binder washout were randomized to tenapanor (*n* = 423) 30 mg bid for 26 weeks or sevelamer carbonate for 52 weeks (*n* = 141). Patients completing 26 weeks of tenapanor were then randomized to tenapanor (*n* = 128) or placebo (*n* = 127). The primary efficacy end-point was the difference in the change in serum phosphorus between tenapanor (+0.22 mg/dl) and placebo (+0.88 mg/dl) during the randomized withdrawal period (*P* = 0.002, intention-to-treat analysis). The mean reduction in serum phosphorus with tenapanor during the 26-week randomized treatment period was 1.4 mg/dl. Among those subjects who achieved a ≥1.2 mg/dl reduction in serum phosphorus with tenapanor during the randomized treatment period, mean serum phosphorus reduction was 2.5 mg/dl over the 26-week period. Drug-related diarrhea resulting in study discontinuation occurred in 17% of patients receiving tenapanor; however, serious adverse events were reported more frequently among patients treated with sevelamer (16%–23% across all study periods) versus tenapanor (11%–17%).

In this issue of Kidney International Reports, Nitta *et al.*^8^ report a study of tenapanor added to phosphate binders among dialysis patients in Japan with refractory hyperphosphatemia. One hundred sixty-nine subjects taking phosphate binders with serum phosphorus 6.1 to 10 mg/dl were randomized 1:1 for 8 weeks to placebo or tenapanor. Their baseline phosphate binder regimen was continued. Study drug was titrated as needed to target serum phosphorus 3.5 to 4.5 mg/dl. To maintain investigator blinding, the central laboratory phosphorus values were masked and reported only as a recommendation to “discontinue study drug” (treatment failure for phosphorus >10 mg/dl), “increase dose” (phosphorus >6.1 mg/dl), “dose can be increased” (phosphorus 4.5–6.0 mg/dl), “maintain dose” (phosphorus 3.5–4.5 mg/d), or “decrease dose” (phosphorus <3.5 mg/dl). The starting tenapanor dose was 5 mg bid with 5 mg up-titration protocol to 30 mg bid as determined by central laboratory recommendations. The primary end-point was the change in serum phosphorus from baseline to 8 weeks of treatment. The least squares change in serum phosphorus from baseline to week 8 was −0.25 mg/dl for the placebo group and −2.00 mg/dl for the tenapanor group (*P* < 0.0001). The mean tenapanor dose was 14.1 mg bid at week 7, the last prescription visit. Adding tenapanor had a similar phosphorus-lowering effect with all phosphate binders, which included calcium-based, lanthanum-based, iron-based, and resin-based compounds. Diarrhea occurred in 63.1% of patients in the tenapanor group and 14.1% of patients in the placebo group; in the tenapanor group, 75.5% of diarrhea events were considered mild and 24.5% were considered moderate; none were considered severe. No patients discontinued the study because of diarrhea. Diarrhea was most frequent among patients taking tenapanor with bixalomer and ferric citrate and least frequent among patients taking tenapanor with sevelamer and sucroferric oxyhydroxide. The investigators noted that the higher the baseline serum phosphorus level, the more tenapanor reduced serum phosphorus. Approximately 40% of subjects in both arms of this study were using laxatives before initiation. The effects of tenapanor on stool consistency may relieve constipation and decrease laxative pill burden.

As of this writing, tenapanor is not approved by the US Food and Drug Administration in the USA for treatment of hyperphosphatemia. The Cardiovascular and Renal Drugs Advisory Committee of the US Food and Drug Administration reviewed the new drug application for tenapanor and voted 9 to 4 that the drug’s benefits outweigh the risks for control of serum phosphorus in adults on dialysis when administered as monotherapy and 10 to 2 that the drug’s benefits outweigh its risks for control of serum phosphorus in adults on dialysis when administered in combination with phosphate binder treatment.^9^ If approved by the US Food and Drug Administration, tenapanor will be the first nonbinder medication available in the United States for treatment of hyperphosphatemia in patients on dialysis. It may be an attractive option for patients whose serum phosphorus remains elevated despite prescription of (but not necessarily adherence to) phosphate binders, and tenapanor’s bid dosing regimen unrelated to meals makes adherence more likely than with binders. A model for step therapy of hyperphosphatemia is presented in Figure 1. Although many patients in clinical trials reported diarrhea with tenapanor, this led to drug discontinuation in relatively few and it may offset the constipation caused by some phosphate binders. The major barrier to tenapanor uptake will probably be its cost. As a new expensive drug, its use will be restricted by payers in the USA. In 2025, when phosphate lowering medications are moved to the bundled payment for dialysis in the USA and the cost of tenapanor and phosphate binders is borne by dialysis providers, it is likely that phosphate lowering algorithms will be established by the dialysis companies. Based on precedent with agents to treat secondary hyperparathyroidism, algorithms for phosphate lowering therapy will likely steer prescribers to use lower cost agents first and will place formulary restrictions on higher cost agents. Until serum phosphorus level in dialysis patients is a metric for payment or public reporting, or there are compelling interventional data demonstrating that lowering serum phosphorus levels closer to the normal range results in improved patient outcomes, it seems likely that payers in the USA will resist the approval of costly tenapanor except for more resistant cases of hyperphosphatemia.

**Figure 1:**
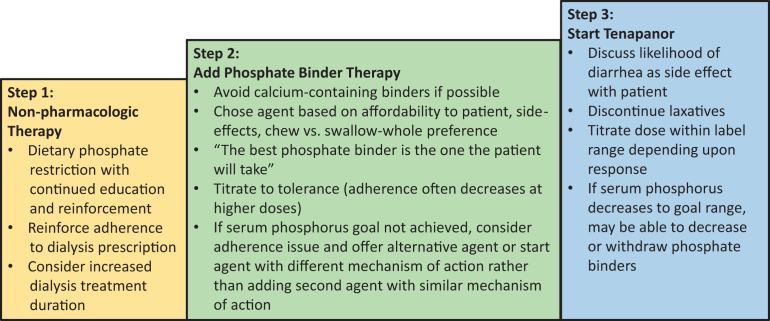
A model for step therapy of hyperphosphatemia in dialysis patients.

## Disclosure

JBW reports personal fees from Akebia, outside the submitted work.

## Supplementary Material

Supplementary File (PDF)

**Supplementary References**.
