# Supplementary material for: Tenapanor as Add-on Treatment for Hyperphosphatemia in Dialysis Patients: Enough Bang for the Buck?
Source: Kidney Int Rep. 2023 Oct 6;8(11):2194–7. doi: 10.1016/j.ekir.2023.10.001 (PMC10658425; doi:10.1016/j.ekir.2023.10.001)
Supplement: Supplementary File (PDF) [file mmc1.pdf]

Supplemental References:

S1. Courivaud C, Devenport A. Phosphate removal by peritoneal dialysis: the effect of transporter status and peritoneal dialysis prescription. *Perit Dial Int.* 2016;36:85–93. doi:10.3747/pdi.2014.00173.

S2. Daugirdas JT. Comparison of measured vs kinetic-model predicted phosphate removal during hemodialysis and hemodiafiltration. *Nephrol Dial Transplant.* 2022;37:2522–2527. doi:10.1093/ndt/gfac223.
